# Autophagy and necroptosis in cisplatin-induced acute kidney injury: Recent advances regarding their role and therapeutic potential

**DOI:** 10.3389/fphar.2023.1103062

**Published:** 2023-01-30

**Authors:** Noha Alassaf, Hala Attia

**Affiliations:** ^1^ Department of Pharmacology and Toxicology, College of Pharmacy, King Saud University, Riyadh, Saudi Arabia; ^2^ Department of Biochemistry, College of Pharmacy, Mansoura University, Mansoura, Egypt

**Keywords:** cisplatin, nephrotoxicity, acute kidney injury, necroptosis, necroinflammation, autophagy, macroautophagy

## Abstract

Cisplatin (CP) is a broad-spectrum antineoplastic agent, used to treat many different types of malignancies due to its high efficacy and low cost. However, its use is largely limited by acute kidney injury (AKI), which, if left untreated, may progress to cause irreversible chronic renal dysfunction. Despite substantial research, the exact mechanisms of CP-induced AKI are still so far unclear and effective therapies are lacking and desperately needed. In recent years, necroptosis, a novel subtype of regulated necrosis, and autophagy, a form of homeostatic housekeeping mechanism have witnessed a burgeoning interest owing to their potential to regulate and alleviate CP-induced AKI. In this review, we elucidate in detail the molecular mechanisms and potential roles of both autophagy and necroptosis in CP-induced AKI. We also explore the potential of targeting these pathways to overcome CP-induced AKI according to recent advances.

## 1 Introduction

Cancer represents a serious health threat and is considered one of the main deadly diseases worldwide (Benyamini et al., 2003; Nguyen et al., 2022). In 2019, approximately 23.6 million new cases and 10 million deaths were associated with cancer (Kocarnik et al., 2022). Cisplatin (CP) is one of the most effective antineoplastic drugs for the management of several solid-organ malignancies including Hodgkin’s and non-Hodgkin’s lymphomas, sarcomas, as well as head, lung, neck, bladder, breast, testicular, ovarian, and cervical cancers (Dasari and Bernard Tchounwou, 2014; Manohar and Leung, 2018). It has been reported that around 50% of all patients are treated with CP in their chemotherapeutic regimens (Chen and Chang, 2019). Interestingly, despite its earlier discovery in 1844 by the Italian chemist, Michele Peyrone, CP did not attract much attention until 1965 (Dasari and Bernard Tchounwou, 2014). During that year, Rosenberg et al. (1965) noticed that the platinum compound released from the platinum electrodes into the growth medium of *Escherichia coli* markedly affects its cellular division and filamentation. This observation has driven a strong wave of preclinical and clinical research for testing its anticancer effect afterward. In 1969, the anticancer effect of CP was evaluated in the sarcoma mouse model (Rosenberg et al., 1969), and by 1971, CP had entered phase I clinical trials and was finally approved by the US Food and Drug Administration for the management of cancers in 1978 (Andrea and Reddy, 2018).

**FIGURE 1:**
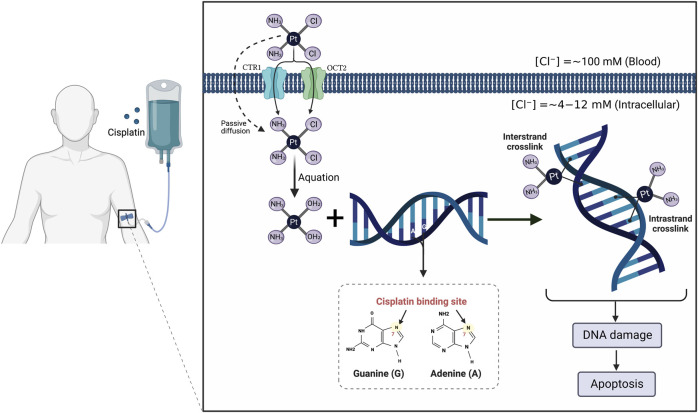
Mechanism of action of cisplatin (CP). The CP structure is composed of a platinum atom in the center, surrounded by two chloride atoms and two ammonia atoms in the cis configuration. Following its intravenous infusion, CP is passed through the cell membrane by passive diffusion or active transport. In the bloodstream, the high chloride concentration (∼100 mM) retains CP in a relatively stable structure and prevents its hydrolysis. Once it enters the cell where the chloride concentration is greatly reduced (∼4–12 mM), the two chlorides are replaced by water molecules in a process termed aquation to produce potent, positively charged electrophilic product. The resulting product covalently binds to the N7 atom of the purine bases, preferentially guanines, in the DNA causing formation of either intrastrand cross-linking when the two organic bases are located on the same DNA strand or interstrand cross-linking is produced if the organic bases are on opposite strands. These cross-links distort the DNA structure and subsequently lead to cell cycle arrest and apoptosis.

**FIGURE 2:**
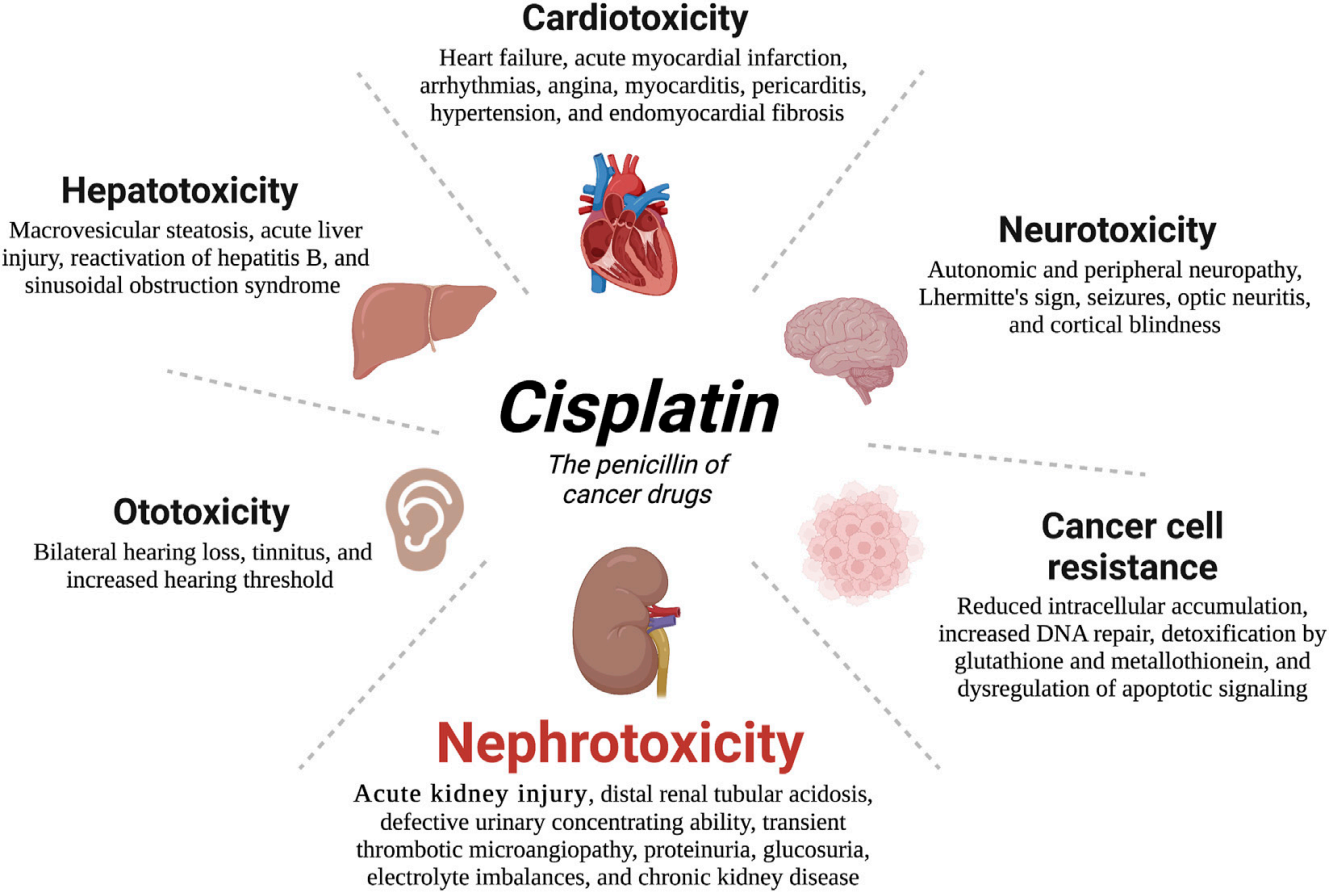
Side effects associated with cisplatin (CP) administration. Numerous deleterious side effects continue to restrict the therapeutic efficacy of CP; with nephrotoxicity especially acute kidney injury being the most serious issue.

It is currently well established that CP mediates its tumoricidal effects through binding to DNA, causing formation of intra- and interstrand cross-links. These cross-links distort the DNA structure and subsequently prevent DNA synthesis and replication in rapidly proliferating cancer cells (Dugbartey et al., 2016) (Figure 1). Despite its efficiency, the clinical use of CP is complicated by multiple side effects such as ototoxicity, neurotoxicity, cardiotoxicity, hepatotoxicity, and cancer cell resistance (Karasawa and Steyger, 2015; Ghosh, 2019) (Figure 2). However, the main serious, dose-limiting, and potentially irreversible side effect of CP is nephrotoxicity (Miller et al., 2010; Hägerström et al., 2019).

CP-induced renal toxicity was originally reported by laboratory animal research in 1971 (Kociba and Sleight, 1971). In humans, CP nephrotoxicity reaches approximately one-third of patients undergoing treatment. Notably, at least 40% of these patients failed to complete the course of therapy owing to renal impairment, which, in turn, negatively affects patients’ survival rate (Shiraishi et al., 2000; Hamroun et al., 2019). CP nephrotoxicity may result in a wide range of manifestations including distal renal tubular acidosis, chronic kidney disease (CKD), defect in urinary concentrating ability, thrombotic microangiopathy, transient proteinuria, glucosuria, hyperuricemia, hypocalcemia, and other electrolyte imbalances, most importantly magnesium and potassium losses (Miller et al., 2010; Oh et al., 2014; Karasawa and Steyger, 2015). However, the most common presentation of CP-induced nephrotoxicity is acute kidney injury (AKI) (Miller et al., 2010), which is characterized by the rapid decline of renal excretory function leading to the accumulation of nitrogenous waste products, water, and electrolytes (Ostermann and Joannidis, 2016; Hu et al., 2021). AKI is a global health problem associated with high mortality which far exceeds those of heart failure, diabetes mellitus, breast, and prostate carcinoma collectively (Lewington et al., 2013).

The pathogenesis of CP-induced AKI is complex and involves multiple molecules and factors. Oxidative stress, mitochondrial dysfunction, inflammation, and apoptosis are all participating in the progression of CP-induced AKI (Xu et al., 2015; Liang et al., 2016). Despite significant progress in elucidation of molecular pathways underlying CP nephrotoxicity, there is no approved treatment currently available for managing such nephrotoxicity except amifostine, which is no longer recommended for this purpose (Sharp and Siskind, 2017). This necessitates further investigation of new molecular targets for preventing or treating this problem. Interestingly, necroptosis and autophagy pathways have emerged recently and have been shown to have a crucial role in CP-induced AKI (Rovetta et al., 2012; Holditch et al., 2019). While many reviews have addressed mechanisms of CP nephrotoxicity, none cover the specific role of both autophagy and necroptosis and their potential for therapeutic intervention. In this review article, we focus on necroptosis and autophagy pathways, giving a comprehensive understanding of their molecular mechanisms, roles in CP-induced AKI, and the potential of targeting necroptosis or autophagy pathways for attenuation of CP-induced AKI. The review also highlights the impact of such interventions on the effectiveness of CP chemotherapy.

## 2 Materials and methods

The PubMed/MEDLINE, Google Scholar, Wiley, and Science Direct databases were used to retrieve relevant publications published until October 2022. We conducted searches using the following search terms: “cisplatin”, “cancer”, “cisplatin nephrotoxicity”, “acute kidney injury”, “cisplatin-induced AKI”, “autophagy”, “AMPK-mTOR-mediated autophagy”, “necroptosis”, “RIPK1/RIPK3/MLKL-mediated necroptosis”, “necroinflammation”, “nephroprotective”, “autophagy activators”, and “necroptosis inhibitors”. The abstracts or full texts of English-written papers were reviewed to determine if they matched the relevant section. Additional studies were obtained from the reference sections of selected studies.

## 3 Renal cellular uptake and biotransformation of cisplatin (CP)

The kidney is the main route of CP excretion, since it has been demonstrated that the concentration of CP in the kidneys is five-fold greater than that in the blood, and therefore such accumulation of the drug within the kidney cells contributes to its nephrotoxicity (Ozkok and Edelstein, 2014; Abdel-Daim et al., 2019). CP is excreted *via* both glomerular filtration and tubular secretion, and it is subsequently accumulated in kidney cells, specifically in the proximal tubular cells. Regarding tubular secretion, CP is actively transported into tubular cells through two membrane transporters, organic cation transporter 2 (OCT2) and copper transporter 1 (CTR1) (Gómez-Sierra et al., 2018).

It is currently believed that CP is actively metabolized in the kidney cells to produce a potent nephrotoxic metabolite (Miller et al., 2010). This pathway begins with the binding of CP to reduced glutathione (GSH) by the action of the glutathione-S-transferase (GST) enzyme in circulation (Gómez-Sierra et al., 2018). The resulting GSH-conjugates are cleaved into a cysteinyl-glycine-conjugate by gamma glutamyltranspeptidase which is localized on the surface of the kidney proximal tubule (Miller et al., 2010; Gómez-Sierra et al., 2018). Aminodipeptidase, also present on the surface of these cells, further metabolized the resulting conjugates to yield cysteine conjugates (Gómez-Sierra et al., 2018). Then, cysteine conjugates are taken up into tubular cells, where they are converted by cysteine-S-conjugate beta-lyase to form highly reactive thiols (Miller et al., 2010). Finally, the formed thiols bind to several cellular proteins and ultimately cause damage and death of renal tubular cells (Zhang and Hanigan, 2003; Peres and da Cunha, 2013).

## 4 Necroptosis in CP-induced acute kidney injury (AKI)

### 4.1 Fundamentals of necroptosis

#### 4.1.1 General overview of necroptosis

The term “programmed cell death” had been commonly used to describe caspase-dependent apoptosis until caspase-independent necrosis was identified (Linkermann et al., 2012). Necroptosis (also called regulated necrosis) is a recently recognized form of cell death that is activated during apoptosis-compromised conditions and shares characteristics of both accidental necrosis and apoptosis (Kaczmarek et al., 2013; Dhuriya and Sharma, 2018; Oliveira et al., 2018). Necroptosis is programmed (like apoptosis) and morphologically characterized by cellular swelling, rupture of the plasma membrane, and organelle dysfunction (like necrosis) (Weinlich et al., 2017; Dhuriya and Sharma, 2018). Additionally, biochemical features of necroptosis may include energy depletion, formation of reactive oxygen species (ROS), and accumulation of calcium (Ca^2+^) (Cho, 2018). Physiologically, necroptotic cell death is emerging as an important process for normal development and host defense against various pathogens (Fulda, 2013; Wu et al., 2014; Nailwal and Chan, 2019). However, dysregulation of necroptosis has been shown to be associated with several diseases and tissue damages including cancer (Dhuriya and Sharma, 2018), ischemia/reperfusion injury (Fulda, 2013), acute pancreatitis (Wang G. et al., 2016), colitis (Hou et al., 2019), drug-induced hepatotoxicity (Ramachandran et al., 2013), atherosclerosis (Coornaert et al., 2018), and neurodegeneration (Oliveira et al., 2018).

#### 4.1.2 Molecular mechanism and regulation of necroptotic cell death

Necroptosis is considered the best and most studied form of regulated necrosis and its molecular mechanism is being thoroughly investigated in recent years (Linkermann, 2016; Li L. et al., 2021). A cascade of several kinases including receptor-interacting protein kinase (RIPK)-1, RIPK3, and mixed lineage kinase domain-like protein (MLKL) has been identified to play a critical role in the regulation of the necroptotic pathway (Wang X. et al., 2018; Shan et al., 2018). Necroptosis can be triggered by multiple endogenous and exogenous stimuli including anticancer agents, metabolic disturbance, ischemia/reperfusion injury, and activation of death receptors such as tumor necrosis factor-α (TNF-α) receptor (TNFR), toll-like receptors (TLRs) particularly TLR3/4, as well as interferon (IFN) receptors (Wang G. et al., 2016; Seifert and Miller, 2017; Dhuriya and Sharma, 2018). Despite diversity of stimuli that responsible for necroptosis initiation, TNF-α remains the best-characterized and probably the most important trigger of necroptosis (Vandenabeele et al., 2010; Shan et al., 2018).

**FIGURE 3:**
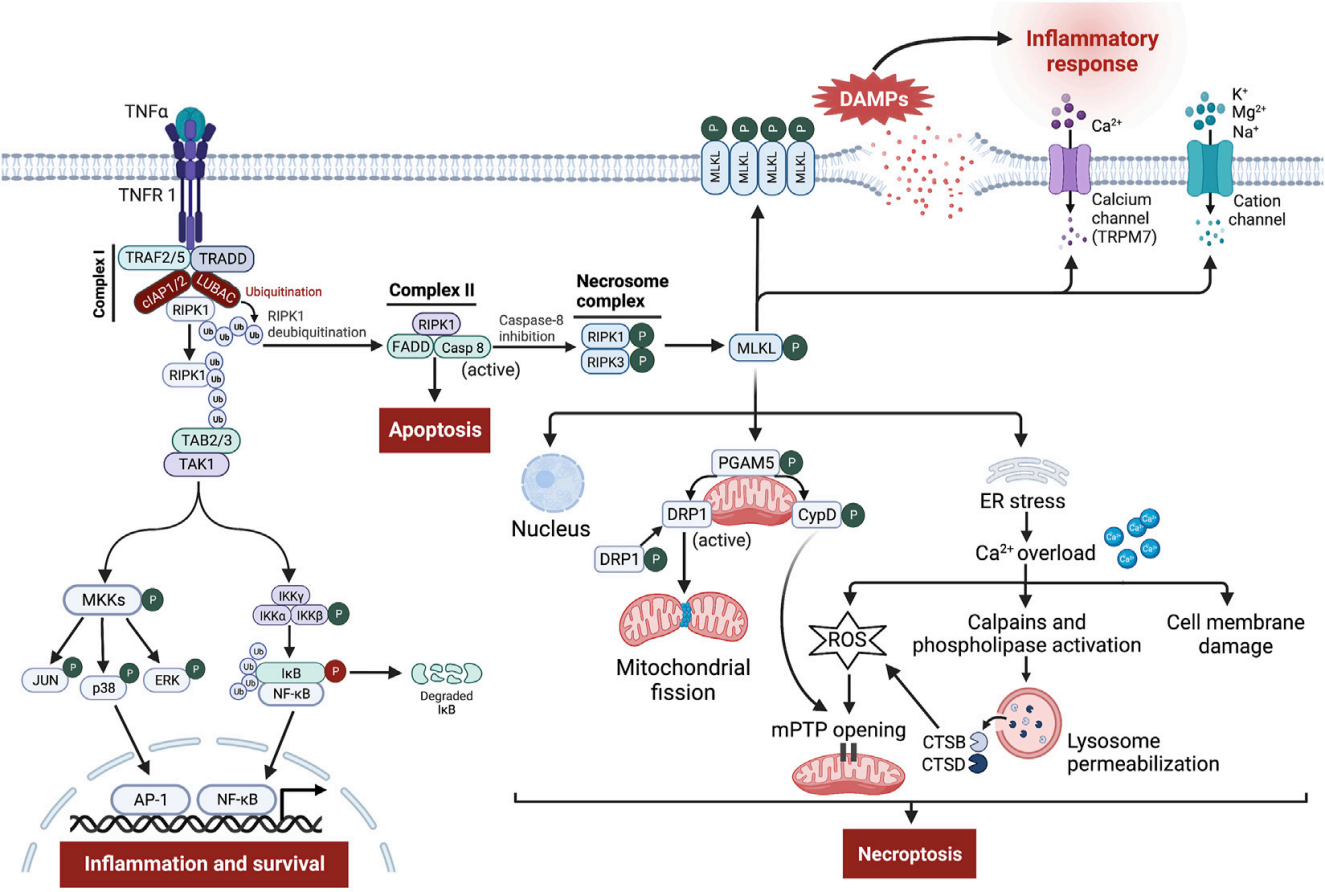
Signaling pathway leading to tumor necrosis factor-α (TNF-α)-receptor 1 (TNFR 1)-mediated necroptosis. Ligation of TNF-α to TNFR1 leads to the generation of the membrane-bound complex I, in which cellular inhibitor of apoptosis proteins (cIAPs) and linear ubiquitin chain assembly complex (LUBAC) polyubiquitinate receptor-interacting protein kinase (RIPK)-1 (RIPK1), directing it towards proteasomal degradation and stabilize complex I, which contributes to inflammation and cell survival through stimulation of nuclear factor kappa B (NF-κB) and mitogen-activated protein kinase (MAPK) pro-survival pathways. However, the deubiquitination of RIPK1 induces the assembly of complex II involving Fas-associated death domain (FADD), RIPK1, and caspase-8. Normally, caspase-8 cleaves RIPK1 and RIPK3, thereby triggering the activation of apoptosis. Nevertheless, upon caspase-8 inhibition, RIPK1 binds to RIPK3 to form the necrosome complex, followed by phosphorylation and formation of mixed lineage kinase domain-like protein (MLKL) oligomers. These oligomers either form pores in the cell membrane or recruit cation channels and calcium (Ca^2+^) channels, namely transient receptor potential melastatin related 7 (TRPM7), thereby allowing the influx of extracellular ions, disrupting osmotic pressure, leading to cell rupture and release of damage-associated molecular patterns (DAMPs) which in turn provoke potent inflammatory responses. Besides, phosphoglycerate mutase family member 5 (PGAM5) on the mitochondrial membrane is recruited and phosphorylated upon necroptosis activation. Once activated, PGAM5 phosphorylates cyclophilin D (CypD) and reverses phosphorylation of dynamin-related protein 1 (Drp1), resulting in their activation where they contribute to mitochondrial fission and mitochondrial permeability transition pore (mPTP) opening, eventually leading to necroptosis. Moreover, a small part of MLKL oligomers translocates into the nucleus, facilitating necroptotic cell death, while other moves to the endoplasmic reticulum (ER), triggering stress response that causes intracellular Ca^2+^ overload. Increased Ca^2+^ concentration executes necroptosis through damage to the cell membrane, overproduction of reactive oxygen species (ROS), which promotes mPTP opening, and finally through stimulation of Ca^2+^-dependent enzymes like calpains and phospholipase leading to permeabilization of lysosome membrane and subsequent release of cathepsin B (CTSB) and D (CTSD) into the cytosol. These enzymes in turn lead to lysosomal dysfunction, and increased oxidative potential, all of which events result in necroptosis.

The binding of TNF-α to its receptor, TNFR1, can lead to either cellular survival, apoptosis, or necroptosis (Seifert and Miller, 2017) (Figure 3). Stimulation of TNFR1 induces certain conformational changes in the receptor, enabling its cytosolic portion to recruit several proteins to form the prosurvival complex I consisting of TNFR-associated death domain (TRADD), TNFR-associated factor 2 and 5 (TRAF2/5), RIPK1, cellular inhibitor of apoptosis proteins 1/2 (cIAP1/2), and linear ubiquitin chain assembly complex (LUBAC) (Nikoletopoulou et al., 2013; Seifert and Miller, 2017; Dhuriya and Sharma, 2018). Within complex I, cIAPs and LUBAC trigger RIPK1 ubiquitination leading to stabilization of complex I, which in turn induces recruitment of TGF-β-activating kinase 1 (TAK1), TAK1-binding protein 2 (TAB2), and TAB3 to generate TAK1-TAB2-TAB3 complex (Liu et al., 2021; Seo et al., 2021). TAK1 phosphorylates and activates the IκB kinase (IKK) complex and MAPK kinases (MKKs), which in turn stimulates nuclear factor kappa B (NF-κB) and activator protein 1 (AP-1) transcriptional activity leading to inflammation and cell survival (Mihaly et al., 2014).

Alternatively, during specific conditions, where complex I is destabilized or the ubiquitination process is inhibited, RIPK1 dissociates from complex I and binds to Fas-associated death domain (FADD) and caspase-8, forming apoptotic complex II that results in induction of TNF-α-induced apoptosis (Seifert and Miller, 2017; Dhuriya and Sharma, 2018; Seo et al., 2021). Within complex II, caspase-8 inhibits necroptosis through proteolytic cleavage of RIPK1 and RIPK3 (Nikoletopoulou et al., 2013). However, exhaustion or reduced activity of caspase-8 shifts cellular fate from apoptosis towards necroptosis (Cho et al., 2009; Leeper, 2016; Seifert and Miller, 2017). Thus, necroptosis acts as a backup pathway for cell death during impairment of caspase-8-dependent apoptosis (Zhang et al., 2016; Dhuriya and Sharma, 2018).

From a molecular point of view, the necroptosis pathway is initiated by auto- and trans-phosphorylation of RIPK1 and RIPK3 at Ser-227, resulting in stimulation of their kinase activity and assembly of a heterodimer protein complex termed necrosome *via* their RIPK homotypic interaction motif domains (Fulda, 2013; Cho, 2018; Dhuriya and Sharma, 2018; Oliveira et al., 2018; Hou et al., 2019). Then, RIPK3 recruits and phosphorylates its substrate MLKL at Thr-357/Ser-358 (Oliveira et al., 2018; Hou et al., 2019). Following phosphorylation, MLKL oligomerizes and a small part of these oligomers moves towards the cell membrane where they mediate their effect in two different ways, either directly by facilitating the formation of pores across the cell membrane mediated through binding to membrane phospholipids like phosphatidylinositol phosphate and cardiolipin or indirectly by increasing influx of ions (Na^+^, K^+^, Mg^2+^, and Ca^2+^) through recruitment of cation channels and Ca^2+^ channels, the transient receptor potential melastatin related 7 (TRPM7), thereby increasing osmotic pressure and eventually leading to cell rupture (vanden Berghe et al., 2016; Seifert and Miller, 2017; Dhuriya and Sharma, 2018; Li L. et al., 2021). Meanwhile, another part of MLKL oligomers translocates into the membranes of various organelles e.g., nucleus, mitochondria, endoplasmic reticulum (ER), and lysosomes, causing their damage *via* necroptotic cell death (Li L. et al., 2021; Jantas and Lasoń, 2021).

### 4.2 Emerging role of necroptosis in CP-induced AKI

Cellular death and inflammation are largely found in the proximal tubular cells, which concur with the main target of CP accumulation (Xu et al., 2015; Ning et al., 2018). In the past, most of the previous research has investigated and characterized apoptosis as the primary cell death in the context of CP nephrotoxicity (Lau, 1999; Cummings and Schnellmann, 2002; Jiang et al., 2004). As a result, strategies targeting the apoptosis mediators have long been studied for the treatment of CP-induced renal injury (Wei et al., 2007; Molitoris et al., 2009; Pabla et al., 2011). However, inhibition of inflammation, tubular necrosis, and necroptosis was sufficient to protect against CP nephrotoxicity even in the presence of apoptosis (Kim et al., 2012; Ozkok et al., 2016; Yoon and Kim, 2018). Moreover, two reports surprisingly demonstrated that suppression of apoptosis alone fails to ameliorate or stop the development of CP-induced nephrotoxicity (Herzog et al., 2012; Sridevi et al., 2013). These data could reflect our limited knowledge of the complex cell death mechanisms involved in the pathogenesis of AKI. Recent advances suggest that in addition to apoptosis, other forms of programmed cell death co-exist and play a significant role in the pathogenesis of CP-induced renal injury. In recent years, tremendous interest has been focused on necroptosis as the most important mechanism of tubular death in CP-induced AKI. The implication of necroptosis in CP-induced AKI was first reported about a decade ago by Tristão’s group (Tristão et al., 2012). Following this observation, several *in vitro* and *in vivo* studies demonstrated that the expression of necroptosis-related proteins (RIPK1, RIPK3, and MLKL) was simultaneously induced following CP administration (Gao et al., 2016; Al-Salam et al., 2021). Interestingly, Xu et al. (2015) reported that the CP-induced tubular damage could be diminished by either knockout or inhibition of RIPK3 or MLKL activity. Later, Gao et al. (2016) and Gwon et al. (2021) further confirmed the implication of necroptosis in CP-induced AKI where pharmacological inhibition of RIPK1/RIPK3/MLKL axis by protocatechuic aldehyde and 6-shogaol, respectively significantly attenuates renal injury. Similarly, pretreatment with novel RIPK1 inhibitors, Cpd-71 and Cpd-2 mitigated CP nephropathy (Wang et al., 2019; Li C. et al., 2022). Likewise, Liu et al. (2022b) found that the novel heat shock protein (Hsp) 90 inhibitor, C-316-1 facilitates ubiquitination and degradation of RIPK1, thereby inhibiting RIPK1-mediated necroptosis and subsequently attenuating CP-induced AKI. Recently, several investigators further emphasized the significance of this contribution by showing that the protective and detrimental effects of various proteins in the CP-induced AKI model were mediated *via* modulation of necroptosis signaling. In this regard, Gao et al. (2018) showed that *in vitro* and *in vivo* enhancement of E-cadherin protein by PPBICA treatment protects against CP-induced AKI by attenuating necroptosis and necroptotic inflammation. In a comparable way, the protective role of Numb and galectin-3 proteins against CP injury has been primarily attributed to the inhibition of tubular necroptosis and/or its related inflammatory response (Liu et al., 2020; Al-Salam et al., 2021). In contrast, another preclinical study found that pyruvate dehydrogenase kinase 4 aggravated CP-induced AKI at least partially by activating necroptosis, while pharmacological or genetic disruption of its activity dampened CP-induced necroptosis, thereby attenuating AKI in mice (Oh et al., 2017). More recently, Yang et al. (2019); Yang et al. (2021) and Wang F. et al. (2022) showed that Smad 2/3 and stratifin proteins play a detrimental role in CP-induced AKI by inducing necroptosis and necroinflammation, and interestingly, targeting these proteins suppressed the necroptosis signaling and thereby alleviating AKI.

#### 4.2.1 Necroptosis and oxidative stress

Intriguingly, how CP would activate tubular necroptosis is still largely unknown, but one possible explanation could be related to the prooxidant activity of CP. Upon administration, CP amplifies the formation of ROS including hydrogen peroxide, hydroxyl radical, and superoxide anion through stimulation of nicotinamide adenine dinucleotide phosphate oxidase enzymes (Chirino et al., 2008; Gómez-Sierra et al., 2018). In addition, CP also affects mitochondrial function by inhibiting activities of various antioxidant enzymes such as GST, GSH-peroxidase, and superoxide dismutase, resulting in an imbalance between oxidant production and endogenous antioxidant defense system, which constitutes oxidative stress (Karasawa and Steyger, 2015). CP may also directly disrupt the mitochondrial respiratory chain leading to the generation of ROS and impairment of mitochondrial function (Pabla and Dong, 2008; Manohar and Leung, 2018). Moreover, during its conversion to a more potent nephrotoxic metabolite, CP binds to GSH, a powerful antioxidant molecule leading to depletion of its cellular levels (Manohar and Leung, 2018). A growing number of studies have shown the reciprocal relationship between ROS and the necroptosis pathway. ROS directly stimulates RIPK1 autophosphorylation, thereby enabling RIPK3 recruitment and necrosome formation (Zhang Y. et al., 2017). In addition, Fan et al. (2022) recently found that increased levels of ROS induced necroptotic cell death in renal tubules, while antioxidants like N-acetylcysteine significantly attenuated this effect. Similarly, inhibition of ROS by butylated hydroxyanisole suppressed activation of TNF-induced necroptosis, suggesting the critical role of ROS in this form of cellular death (Lin et al., 2004). Conversely, following necroptotic stimuli, RIPK3 activates several metabolic regulatory enzymes, thereby increasing energy metabolism and ROS, which in turn further enhance necroptosis activation (Zhang et al., 2009). Similarly, Yang Z. et al. (2018) showed that the necrosome-containing RIPK3 and MLKL enhanced aerobic respiration, which in turn increased the production of mitochondrial ROS. These ROS act in a positive feedback circle to induce necroptosis. This connection was also confirmed by Zhu et al.’s (2018) group who found that upregulation of RIPK3 triggers ER stress which subsequently increased Ca^2+^ overload and xanthine oxidase activity leading to overproduction of ROS, all of which was reduced by RIPK3 deletion. Collectively, these data may suggest that ROS outbursts induced by CP treatment may represent a critical mediator in regulating necroptosis induction.

#### 4.2.2 Necroptosis and inflammation

Notably, necroptosis rather than apoptosis provokes the inflammatory reaction in AKI (Meng et al., 2018). Dying of proximal tubular cells by necroptosis induced the release of endogenous components like damage-associated molecular patterns (DAMPs) including high-mobility group box 1, Hsps, uric acid, interleukin-33, *etc.*, which in turn activate downstream inflammatory signaling like TLRs signaling, thereby triggering robust inflammatory responses (Gao et al., 2018; Seo et al., 2021). Previous studies using genetic or small-molecule inhibitors of necroptosis *via* RIPK1/RIPK3/MLKL axis have given accumulating evidence regarding the essential role of necroptosis in ameliorating multi-organ inflammation such as skin inflammation (Bonnet et al., 2011), vascular inflammation (Lin et al., 2013), and inflammation of the pancreas (Wu et al., 2013), liver (Gautheron et al., 2014), and intestine (Welz et al., 2011; Lee et al., 2020). A similar situation was observed in kidney research, where blockade of necroptosis signaling greatly attenuates inflammatory response associated with tubulointerstitial injury and nephrotoxic nephritis (Xiao et al., 2017; Hill et al., 2018). In addition, Mulay et al. (2016a) also demonstrated that RIPK3 and MLKL-deficient mice were protected from tubular injury and interstitial inflammation induced by crystal deposition. Furthermore, necroptotic inflammation is also considered an important driving factor for kidney graft failure. The proinflammatory DAMPs molecules released by necroptosis in renal allografts triggered the inflammatory injury, thereby accelerating allograft rejection. Conversely, RIPK3 deficiency under this setting prevented necroptosis and the subsequent release of DAMPs, thereby prolonging allograft survival following transplantation (Lau et al., 2013). Besides the direct cytotoxic and prooxidant effects of CP treatment, necroptosis could be induced indirectly through the combined action of cytokines, TNF-α, TNF-related weak inducer of apoptosis, and IFN-γ, which were upregulated upon CP administration. Interestingly, suppression of the necroptotic pathway significantly diminished cytokines upregulation and attenuated the inflammatory response in the CP-induced AKI model (Xu et al., 2015). These data indicate the positive feedback circle involving necroptosis and inflammation during CP-induced AKI, where induction of one factor activates another. Similarly in myocardial infarction, stroke, and acute tubular necrosis, the inflammatory response which is activated after initial insult further augmented necroptotic cell death (Mulay et al., 2016b). This augmentation occurs either directly through the stimulation of TNFR1 by TNF released from necrotic cells or indirectly through the recruitment and activation of leukocytes including macrophages, neutrophils, lymphocytes, and other proinflammatory cells that contribute to tissue injury (Linkermann et al., 2014; Anders, 2018).

#### 4.2.3 Necroptosis in AKI–CKD transition

Importantly, recent data indicated that the reciprocal enhancement between necroptosis and inflammation in this auto-amplification loop could further promote kidney damage, leading to fibrosis and chronic organ failure. Under the conditions of unilateral ureteral obstruction and renal ischemia/reperfusion injury, tubular necroptosis is markedly upregulated, which in turn promotes NOD-like receptor protein 3 (NLRP3) inflammasome activation leading to renal fibrosis. Whereas genetic or pharmacologic inhibition of the necroptosis axis prevented activation of necroinflammation and subsequent development of renal fibrogenesis (Chen et al., 2018; Jiang et al., 2022; Xuan et al., 2022), implying a relationship between necroinflammation and renal fibrosis. Most importantly, in the CP-induced AKI model, Landau et al. (2019) found that continuous activation of regulated necrosis following CP treatment was shown to be the most important factor that drives the transition and progression of AKI to chronic irreparable kidney disease. These findings suggest involvement of necroptosis signaling in both CP-induced acute and chronic kidney diseases, further making necroptosis a valuable target for therapeutic intervention during CP chemotherapy.

#### 4.2.4 Targeting necroptosis in CP-induced AKI

Given the central role of the necroptotic pathway and its associated inflammation in the pathogenesis of CP-induced AKI along with their potential role in AKI-to-CKD transition, multiple agents with anti-necroptotic activity were tested in the CP-induced AKI model. However, currently available agents are still limited (Table 1) and despite their considerable merits, multiple concerns were raised concerning the use of these inhibitors, specifically those targeting RIPK1. The first issue associated with RIPK1 inhibitors like wogonin and PPBICA is their low water solubility and limited bioavailability (Baek et al., 2018; Liu et al., 2022), together with cardiovascular problems of hydrogen sulfide (Beck and Pfeilschifter, 2022), all of which greatly limits the clinical application and translation of these agents. Likewise, the first identified RIPK1 inhibitor, Necrostatin-1 has somehow limited utility due to low solubility, short half-life of about 1 h along with narrow structure–activity relationship profile (Degterev et al., 2008; Oliveira et al., 2018; Wang et al., 2019). Another critical issue that was raised regarding Necrostatin-1 is its non-specificity. For example, it was shown that Necrostatin-1 blocked apoptosis and partially inhibits the two human kinases, PAK1 and PKAcα (Biton and Ashkenazi, 2011; Hill et al., 2018). Shortly thereafter, Takahashi N. et al. (2012) demonstrated that Necrostatin-1 also inhibits the activity of other enzymes as indoleamine 2,3-dioxygenase, which plays an important role in the innate and adaptive immune systems. Thus, this could necessitate careful interpretation of its biological effect *in vivo* and suggests importance of targeting and assessing other more specific downstream mediators, i.e., RIPK3 and MLKL for suppressing necroptosis. Other factors that should be taken into account are the dose and duration of treatment for these inhibitors. Necroptosis is usually induced after the administration of high doses of CP or following its long-time exposure at low concentrations. Therefore, the ability of these agents to produce notable protective effects and ameliorate renal injury is expected to observe several days after treatment (Deng et al., 2021).

**TABLE 1:** Summary of molecules targeting necroptosis in CP-induced AKI.

Compound	Classification	Experimental evidence		Molecular target	Effects on CP-induced AKI	Use of necroptosis inhibitor or genetic knockout of necroptotic molecules	Effect of necroptosis inhibitor on CP’s chemosensitivity	References
		*In vitro*	*In vivo*					
Wogonin	Natural monoflavonoid	HK2	C57BL/6 mice	RIPK1	Inhibition of necroptosis suppresses renal inflammation and tubular death and protects against AKI	Reversed protection	Combining wogonin with CP significantly promoted the anticancer effect of CP in hepatoma HepG2 cells, but did not affect proliferation of hepatoma BEL-7402 and gastric cancer SGC-7901 cell lines	Meng et al. (2018)
Necrostatin-1	RIPK1 kinase inhibitor	n/a	C57BL/6 mice	RIPK1	Inhibition of necroptosis alleviates AKI	n/a	In combination, Necrostatin-1 significantly attenuated CP-induced cancer cell apoptosis in KYSE510, but not in KYSE410 esophageal cancer cell lines	Ning et al. (2018); Zhang et al. (2021b)
7-Hydroxycoumarin (umbelliferone)	Coumarin derivative	HK-2	C57BL/6 mice	RIPK1, RIPK3, and p-MLKL	Inhibition of necroptosis prevents the development of necroinflammation and AKI	n/a	Coadministration of umbelliferone with CP significantly increased cytotoxicity of CP in HL-60 leukemia and HeLa cervical cancer cell lines	Wu et al. (2020b); Ali et al. (2021)
Hydrogen sulfide	Gaseous signal molecule	n/a	dogs (beagles)	RIPK1 and RIPK3	Inhibition of necroptosis reduces expressions of pro-inflammatory factors and mitigates AKI	n/a	The combination of two agents had no impact on antitumor activity of CP in liver cancer HepG2 and breast cancer MCF7 cells	Cao et al. (2018); Wang et al. (2022b)
Protocatechuic aldehyde	Phenolic aldehyde	HK2	Mice	RIPK1, RIPK3, and p-MLKL	Inhibition of necroptosis attenuates renal inflammation and AKI	n/a	Coadministration of protocatechuic aldehyde with CP did not alter the anticancer efficiency of CP in malignant glioma U87, liver cancer SMCC-7721 and BEL-7402 cell lines	Gao et al. (2016)
C-316-1	Hsp90 inhibitor	HK2	C57BL/6J mice	RIPK1	Inhibition of necroptosis ameliorates inflammatory response and AKI	n/a	n/a	Liu et al. (2022)
Compound-71	Novel RIPK1 inhibitor	HK2	C57BL/6J mice	RIPK1	Inhibition of necroptosis reduces oxidative stress and inflammation induced by CP and protects against AKI	n/a	n/a	Wang et al. (2019)
6-Shogaol	Active compound of ginger	n/a	C57BL/6N mice	RIPK1, RIPK3, and p-MLKL	Inhibition of necroptosis attenuates CP-induced inflammation and AKI	n/a	n/a	Gwon et al. (2021)
Kahweol	Natural coffee-specific diterpene	n/a	C57BL/6N mice	RIPK1, RIPK3, and p-MLKL	Inhibition of necroptosis attenuates inflammatory responses and AKI	n/a	n/a	Kim et al. (2020)
Melatonin	Pineal hormone	TCMK-1	C57BL/6N	RIPK1 and RIPK3	Inhibition of necroptosis ameliorates CP-induced inflammation and AKI	n/a	Combined treatment of melatonin and CP reinforced the effectiveness of CP against Cal-27 and SCC-9 head and neck carcinoma cell lines	Fernandez-Gil et al. (2019); Kim et al. (2019)
PPBICA	Small molecule discovered by high-throughput screening	mTECs	C57/BL mice	RIPK1 and RIPK3	Inhibition of necroptosis attenuated CP-induced inflammatory response and AKI	Attenuated protection	n/a	Gao et al. (2018)

Abbreviations: CP, cisplatin; AKI, acute kidney injury; HK2, human tubular epithelial cells; RIPK1, receptor-interacting protein kinase 1; RIPK3, receptor-interacting protein kinase 3; p-MLKL, phosphorylated mixed lineage kinase domain-like protein; Hsp90, heat shock protein 90; TCMK-1, mouse renal tubular epithelial cells; mTECs, mouse kidney proximal tubular epithelial cell line, ↑; increased, ↓; decreased, n/a; not assessed.

#### 4.2.5 Interplay between necroptosis and apoptosis in CP-induced AKI

Undoubtedly, both apoptotic and necroptotic cell death coexist in the pathophysiological course of AKI (Wang S. et al., 2016). In *in vivo* model of CP-induced nephrotoxicity, both apoptotic and necroptotic cell death pathways were shown to be concomitantly induced in kidney tubules following CP treatment; whereas *in vitro* various forms of regulated cell death are activated at different stages of renal injury depending on CP concentration (Kim et al., 2020; Deng et al., 2021; Gwon et al., 2021). Interestingly, these various forms, specifically necroptosis and apoptosis could interplay at various cellular and molecular levels and thus could mutually influence each other. A recent study by Zhang S. et al. (2019) revealed the important role of RIPK3 in mediating renal tubular cell apoptosis in endotoxin/sepsis-induced AKI. Reducing RIPK3 expression or inhibiting its activity significantly reversed the elevation of cleaved caspase-3 and proapoptotic protein Bax, thereby ameliorating AKI. Furthermore, this interplay is well exemplified by the findings of previous studies that showed that administration of pan-caspase inhibitor, zVAD-fmk potentially facilitated RIPK-mediated necroptosis in several renal models including AKI induced by CP (Linkermann et al., 2013; Tristão et al., 2016; Zhu et al., 2016; Zhu et al., 2018). Therefore, although the apoptosis pathway has been considered an important target for the attenuation of CP-induced AKI for many years, inhibition of apoptosis only could paradoxically sensitize tubular death through necroptosis signaling. Therefore, optimal protection against such injury may necessitate the antagonizing of both pathways.

In this regard, a previous report by Linkermann et al. (2013) compared RIPK3- to caspase 8/RIPK3–double knockout mice in the CP-induced AKI model. The authors found that caspase 8/RIPK3–double knockout mice showed a significant improvement in survival kinetics in this model, indicating that combined blockade of necroptotic and apoptotic pathways could provide additional protection. Later, multiple studies have further highlighted this concept by demonstrating that several agents attenuate CP-induced renal injury through dual suppression of both CP-induced apoptosis and necroptosis processes (Kim et al., 2019; Kim et al., 2020; Gwon et al., 2021). Recently, the impact of such synergism has been investigated and confirmed to be effective in mitigating CP-induced nephrotoxicity. Tristão’s group confirmed in 2 publications that the simultaneous use of apoptotic inhibitor, Z-VAD-fmk, and necroptotic inhibitor, necrostatin-1 synergistically attenuates CP nephrotoxicity (Tristão et al., 2012; 2016). Similar to the CP-induced AKI model, necroptosis inhibitors synergize apoptosis inhibitors to attenuate renal injury in the rat subtotal nephrectomy model (Zhu et al., 2016).

## 5 Autophagy in CP-induced AKI

### 5.1 Introduction of autophagy pathway

#### 5.1.1 An overview of autophagy and its main types

In the late 1960s, Christian de Duve, the Nobel Laureate, first introduced the concept of autophagy. The expression derives from Greek words which originally mean self-eating (Deter and de Duve, 1967; Klionsky, 2008; Glick et al., 2010). Autophagy is a highly conserved and tightly controlled cellular process responsible for the degradation of the damaged cytoplasmic organelles, misfolded proteins, and other macromolecules within the lysosome (Miller et al., 2010; Levine and Klionsky, 2017). Once degraded, the resulting autophagic products including amino acids, fatty acids, and sugars are recycled for energy production and protein synthesis. In this manner, autophagy maintains cellular homeostasis during periods of stress and starvation (Kaushal, 2012; Sciarretta et al., 2018). In response to DNA damage or oxidative stress, autophagy is strongly induced, allowing for cell survival through utilization of autophagic products for energy production and protein synthesis (Levine and Yuan, 2005). In contrast, inhibition of autophagy under such conditions resulted in cellular damage and apoptosis (Amaravadi et al., 2007).

Various types of autophagy have been recognized in mammalian cells, involving i) macroautophagy, ii) microautophagy and iii) chaperone-mediated autophagy, all of which act through the lysosome-dependent pathway but differ in the machinery used to deliver autophagic cargo to the lumen of lysosomes (Ravikumar et al., 2009; Glick et al., 2010; Kaushik et al., 2011) (Table 2).

**TABLE 2:** The main characteristics of different types of autophagy.

	Macroautophagy	Microautophagy	Chaperone-mediated autophagy	References
Process selectivity	Can be selective or non-selective	Can be selective or non-selective	Selective	Feng et al. (2014); Napolitano et al. (2015)
Cargo	Includes proteins and organelles	Includes proteins and organelles	Includes proteins only	Massey et al. (2004)
Mechanism of cargo uptake	Involves autophagosome formation	Occurs by lysosomal membrane invagination	Occurs through lysosomal receptor/substrate interaction	Massey et al. (2004); Feng et al. (2014)

The most common type of autophagy is macroautophagy (henceforth referred to as autophagy). It is a process in which cells sequester the autophagic cargoes within double-membrane vesicular structures, named autophagosomes, which finally fuse with lysosomes, where sequestered materials are degraded and recycled for reuse (Yang et al., 2008; Ravikumar et al., 2009; Levine and Klionsky, 2017; Danieli and Martens, 2018). On the other hand, in microautophagy, lysosomes can directly sequester cytosolic components by invagination of the lysosomal membrane (Ravikumar et al., 2009). It should be noted that the sequestration process in the macro-and micro-types can be either non-selective, which primarily occurs during nutrient starvation, or selective when targeting specific cargoes towards autophagosomes. These cargoes may include protein aggregates (named aggrephagy), damaged organelles (e.g., mitochondria, so-called mitophagy), and invasive pathogens (termed xenophagy) (Mizushima et al., 2008; Glick et al., 2010; Kaushik et al., 2011; Hou et al., 2013; Levine and Klionsky, 2017). In contrast to the former two types, chaperone-mediated autophagy is responsible for selective degradation of specific soluble proteins bearing a particular pentapeptide motif named “KFERQ” (Dice, 2007). This motif is detected by the Hsp70 chaperone complex, which allows the interaction between this substrate protein and the lysosomal receptor. Subsequently, the substrate unfolds and crosses the lysosomal membrane for degradation (Ravikumar et al., 2009; Cuervo, 2011; Kaushik et al., 2011).

#### 5.1.2 Molecular mechanism of autophagy pathway

Our advanced understanding of autophagic machinery and its regulation is attributed to a series of investigations conducted in molecular biology laboratories. These investigations led to the discovery of at least 30 genes named autophagy-related genes (Atg) that are essential for the execution of each step of the autophagic pathway (Ravikumar et al., 2009). The autophagic pathway moves through multiple steps, including the formation of the phagophore, autophagosome, and eventually, autolysosome where degradation of sequestered substances occurs (Yang and Klionsky, 2010; Liu et al., 2017).

The formation of phagophore (also called isolation membrane or autophagosome precursor) occurs primarily at the ER which ultimately elongates and encloses forming the autophagosome structure (Ravikumar et al., 2009). The phagophore, and hence autophagosome formation requires the generation of an initiation complex named Unc51-like kinase 1 (ULK1) complex composed of ULK1, Atg13, FIP200, and Atg101 (Yang et al., 2015; Chen et al., 2016). The ULK1 phosphorylates activating molecule in Beclin1-regulated autophagy protein 1 (AMBRA1) leading to translocation of Bcl-2-interacting myosin-like coiled-coil protein (Beclin-1) towards the ER. The Beclin-1, in turn, forms a complex with vacuolar protein sorting (VPS)-34, VPS15, and Atg14L. In this complex, the lipid kinase, VPS34 phosphorylates phosphatidylinositol into phosphatidylinositol 3-phosphate, which acts as a recruitment signal for multiple proteins that are necessary for the nucleation of phagophore (Karakaş and Gözüaçik, 2014; Lee, 2014; Ryter and Choi, 2015; Mercer et al., 2018; Pavlinov et al., 2020).

**FIGURE 4:**
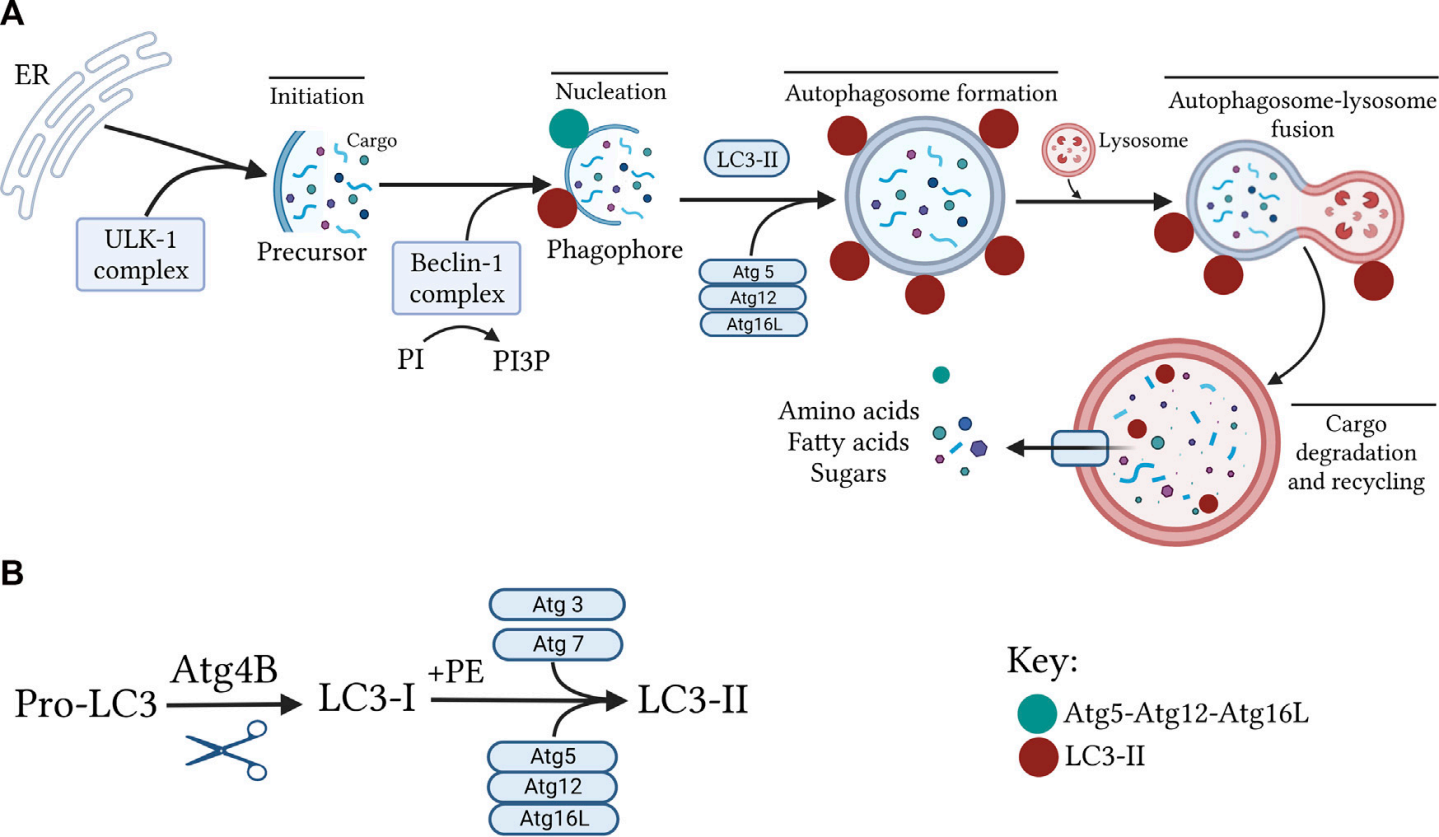
Molecular mechanism of the autophagy pathway. **(A)** the scheme depicts the sequential steps involved in the autophagy process. This process starts with the formation of isolation membrane by the Unc51-like kinase 1 (ULK1) complex primarily at the endoplasmic reticulum (ER) followed by nucleation of this membrane to produce the cup-shaped phagophore. Subsequently, the Atg5–Atg12-Atg16L complex along with processed light chain-II (LC3-II) extended this phagophore to encase autophagic cargo in a double-membraned autophagosome. The loaded autophagosome then merges with the lysosome for breaking down of infused cargo and recycling. Notably, the Atg5–Atg12-Atg16L complex is dissociated from the autophagosomal membrane while LC3-II continues to attach to the mature autophagosomes until vesicle degradation, making it a specific and reliable autophagosomal marker. **(B)** LC3 processing. Upon activation of autophagy, pro-LC3 is first cleaved by Atg4B to produce LC3-I. This is followed by lipidation of LC3-I with phosphatidylethanolamine (PE) to generate LC3-II with the help of Atg7 and Atg3 as well as the Atg5-Atg12-Atg16L complex.

The elongation and closure of the autophagosomal membrane are regulated by two ubiquitylation-like systems; the Atg5-Atg12-Atg16L complex and light chain 3 (LC3) (Ravikumar et al., 2009; Karakaş and Gözüaçik, 2014). On the elongating membrane, pro-LC3 is first cleaved to produce the cytosolic form of LC3 (LC3-I). Then, LC3-I is combined with phosphatidylethanolamine to generate the membrane-bound form of LC3 (LC3-II) by the action of many Atgs including Atg7, Atg3, and Atg5-Atg12-Atg16L complex (Ravikumar et al., 2009; Klapan et al., 2022). Upon closure, mature autophagosome engulfs the autophagic cargoes and subsequently fuses with lysosomes. Finally, the autophagic cargoes are metabolized by hydrolytic enzymes of lysosomes, and degraded contents are returned into the cytoplasm through lysosomal efflux permeases for reuse (Jing and Lim, 2012; Jin and Klionsky, 2014; Xu et al., 2020) (Figure 4).

#### 5.1.3 Regulation of autophagy in mammalian cells

Multiple kinases are responsible for regulation of mammalian autophagy, among them, the mechanistic target of rapamycin (mTOR) and adenosine monophosphate (AMP)-activated protein kinase (AMPK) play important and antagonistic roles (Tamargo-Gómez and Mariño, 2018).

The mTOR is a serine/threonine protein kinase, belonging to the phosphatidylinositol 3-kinase-related kinase family and it is widely known for its negative regulation of the autophagy process (Rabanal-Ruiz et al., 2017; Yuan et al., 2018). The mTOR exists in two complexes called mTOR complex 1 (mTORC1) and mTOR complex 2 (mTORC2) (Hall, 2008). These two complexes vary in their sensitivities to rapamycin, structures, and functions (Yu and Cui, 2016). There is a general consensus that the mTORC1 is the main modulator of autophagy, whereas mTORC2 primarily controls cytoskeleton reorganization (Karakaş and Gözüaçik, 2014).

Under the rich nutrient state, mTORC1 binds to the ULK1 complex and inhibits the initiation of autophagy through inhibitory phosphorylation of ULK1 at Ser-757 (Kim et al., 2011; Jain et al., 2013; Lee, 2014). However, suppression of mTORC1 activity either physiologically by starvation, stress, and certain immunological signals or pharmacologically by rapamycin triggers its dissociation from the ULK1 complex, thus enabling ULK1 to induce autophagy (Ravikumar et al., 2009; Lee, 2014; Liu et al., 2017). It is important to note that, mTOR can directly suppress the activity of Atg13 through inhibitory phosphorylation on Ser-258, thereby preventing association and activation of the ULK1 complex (Jain et al., 2013; Puente et al., 2016). Moreover, it has been found that mTOR may also phosphorylate AMBRA1 to further disrupt the autophagic pathway (Nazio et al., 2013).

Recently, mTOR has also been shown to regulate the autophagy process by controlling the subcellular localization of transcription factor EB (TFEB) (Tong and Song, 2015). The TFEB is widely known for its roles in lipid catabolism, cell metabolism, and lysosomal biogenesis (Soukas and Zhou, 2019). In response to nutrient abundance, mTORC1 phosphorylates TFEB at the lysosomal membrane, thereby inhibiting its migration to the nucleus and promoting its retention in the cytosol in an inactive state (Tong and Song, 2015; Zhitomirsky et al., 2018). In contrast, under conditions of nutrient deprivation, oxidative stress, and lysosomal dysfunction, inhibition of mTOR activity and concurrent activation of calcineurin triggers TFEB dephosphorylation (Medina et al., 2015; Tong and Song, 2015; Napolitano et al., 2018). During these conditions, the lysosome releases Ca^2+^, which in turn activates the Ca^2+^-activated phosphatase, calcineurin (Zhang et al., 2020). Upon activation, calcineurin dephosphorylates and stimulates TFEB, which in turn translocates into the nucleus where it binds to the coordinated lysosomal expression and regulation (CLEAR) sequence to upregulate expression of several genes involved in the autophagic pathway (Tong and Song, 2015; Slade and Pulinilkunnil, 2017; Sciarretta et al., 2018).

**FIGURE 5:**
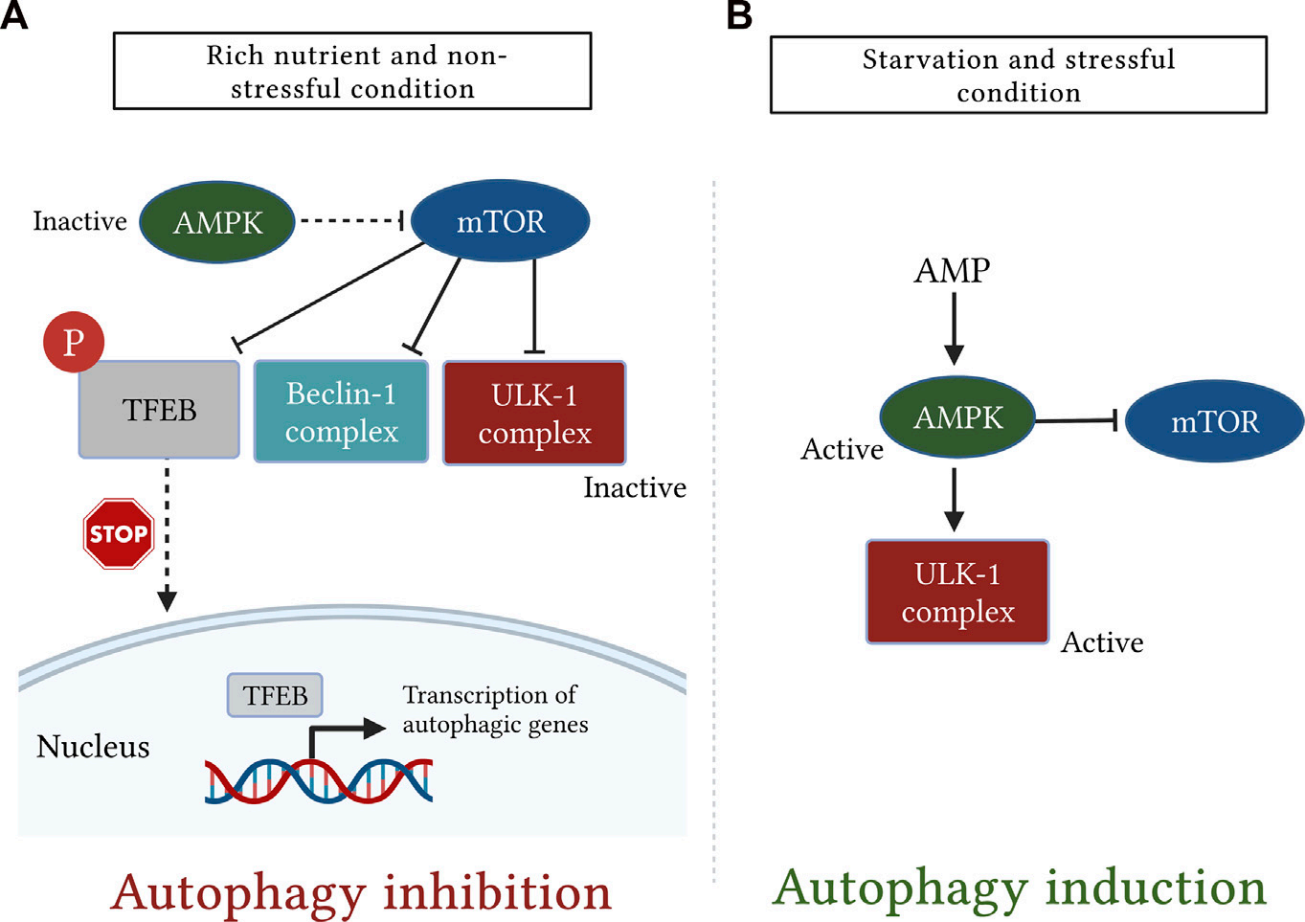
A simplistic diagram showing the antagonistic role of the mechanistic target of rapamycin (mTOR) and AMP-activated protein kinase (AMPK) in regulation of mammalian autophagy. The cellular energy status is closely monitored by the two kinases, mTOR and AMPK. **(A)**: under rich nutrient state and absence of environmental stress, mTOR is activated and suppresses autophagy by disrupting the activation of Unc51-like kinase (ULK1) and Bcl-2-interacting myosin-like coiled-coil protein (Beclin-1) complexes. mTOR also inhibits autophagy by phosphorylating transcription factor EB (TFEB), which, in turn, sequesters TFEB in the cytosol and prevents it from being translocated into the nucleus. **(B)**: Conversely, following starvation or stress response, AMPK is activated and induces autophagy either by direct phosphorylation of ULK1 or indirectly through downregulation of mTOR signaling.

The AMPK is another important regulator of autophagic machinery (Karakaş and Gözüaçik, 2014). The AMPK has a unique heterotrimeric structure consisting of one catalytic subunit (α) along with two regulatory subunits (β and γ) (Garcia and Shaw, 2017). It is tightly regulated by ATP to AMP ratio which is greatly reduced during glucose deprivation leading to AMPK activation (Jung et al., 2010). During the nutrient deprivation state, AMP molecules bind to the AMPK γ subunit and cause certain conformational changes in the heterotrimeric complex, thereby exposing Thr-172 on the catalytic α subunit for phosphorylation as well as activation by liver kinase B1 (Zaha and Young, 2012). Importantly, AMPK stimulates the autophagic pathway through several mechanisms. Among these mechanisms, AMPK suppresses mTOR signaling either directly *via* inactivation of regulatory-associated protein of mTOR or indirectly by activation of its downstream negative regulator tuberous sclerosis complex-1 and -2 (Jung et al., 2010). Moreover, AMPK also stimulates autophagy independently of the mTOR signaling pathway through activating phosphorylation of ULK1 (Kim et al., 2011) (Figure 5).

### 5.2 Role of enhancing autophagy in CP-induced AKI

There is mounting evidence suggesting that basal autophagic activity in the kidney is clearly required for the maintenance and function of the proximal tubule (Kimura et al., 2011; Havasi and Dong, 2016; Zhu et al., 2020). Recently, autophagy has attracted unprecedented interest due to its importance in several renal diseases including AKI induced by CP (Pallet et al., 2008; Ding et al., 2014; Li T. et al., 2017; Nam et al., 2019; Shen et al., 2021). It is currently known that CP induces oxidative stress and DNA damage leading to autophagy induction (Yang et al., 2008; Takahashi A. et al., 2012). In contrast to CP-induced apoptosis which is going through the preapoptotic lag phase (Kaushal et al., 2001; Cummings and Schnellmann, 2002), it has been demonstrated that treatment of kidney epithelial cells with CP immediately and transiently activated autophagy within a few hours of CP administration (Periyasamy-Thandavan et al., 2008; Yang et al., 2008). Activation of autophagy during the initial phase of CP injury could provide a suitable environment for maintaining cell homeostasis before reaching the CP-induced apoptosis threshold (Herzog et al., 2012). However, importantly, high concentrations (≥50 μM) or prolonged treatment of CP have significantly reduced autophagy, thereby dominating apoptotic cell death in kidney cells (Rovetta et al., 2012; Zhang D. et al., 2017).

The cytoprotective and prosurvival role of autophagy in CP-induced AKI has been proved in many pharmacological and Atg-gene knockout studies (Periyasamy-Thandavan et al., 2008; Jiang et al., 2012; Rovetta et al., 2012; Liu J. et al., 2018). It has been reported that stimulation of autophagy after exposure to CP delayed caspase activation and increased the survival of renal cells (Yang et al., 2008; Liu et al., 2016). It was also found that overexpression of autophagic proteins Atg5 or beclin-1 blocked the activation of caspase-3 and cell death induced by CP (Herzog et al., 2012). In contrast, inhibition of autophagy either by chloroquine or knockout of proximal tubule-specific Atg7 worsens kidney function (Jiang et al., 2012). Similarly, Takahashi A. et al. (2012) found that deficiency of autophagy in proximal tubule cells leads to an increase in tubular injury, p53 activation, and protein aggregation in the CP-induced AKI model. In addition, CP exposure also induced PTEN-induced kinase protein 1 (PINK1) and Parkin, the two mediators of mitochondrial autophagy that were recently involved in CP nephrotoxicity. Knockout of either Parkin or PINK1 suppressed mitochondrial autophagy and aggravated kidney injury (Wang Y. et al., 2018).

The role of autophagy in CP-induced AKI was further proposed in subsequent studies. Periyasamy-Thandavan et al. (2008) and Zhao et al. (2017) pointed out that pharmacological inhibition of autophagy by 3-methyladenine enhanced mitochondrial dysfunction and tubular apoptosis during CP treatment. Furthermore, another study indicated that the nephroprotective effect mediated through TLR-2 is in part attributed to activation of autophagy and knockout of TLR2 reduced expression of autophagy proteins and exacerbated renal dysfunction in the CP-induced AKI model (Andrade-Silva et al., 2018). Likewise, it was also demonstrated that the nephroprotection rendered by hypoxia-inducible factor 1-alpha protein on CP-induced AKI was associated with autophagy upregulation (Liang et al., 2017). Consistently, the study by Tang et al. (2018) showed that inhibition of histone deacetylase 6 attenuated CP-induced AKI partially through activation of autophagy. In line with this, Minocha et al. (2019) found that amniotic fluid stem cells exerted their protective effects against CP-induced AKI by upregulating autophagy. Recently, Lynch et al. (2020) demonstrated that the inhibitory effect of peroxisome proliferator-activated receptor gamma coactivator 1α (PGC1α) on ROS induced by CP was mediated through TFEB-dependent autophagy. More recently, Sears et al. (2022) demonstrated that the loss of neutral ceramidase, an enzyme responsible for the metabolism of sphingolipid prevents the development of CP-indued AKI by enhancing basal autophagic activity in the kidney.

#### 5.2.1 The potential mechanisms underlying autophagy protection

The exact mechanisms behind the cytoprotective role of the autophagy pathway are not yet completely understood. However, multiple mechanisms have been suggested, including the following: i) Through degradation of different cellular components, autophagy allows for the turnover of resulting substrates for energy production and protein formation, which is considered essential for the preservation of cellular bioenergetics during AKI (Jiang et al., 2012). ii) Autophagy eliminates depolarized mitochondria, dysfunctional or damaged organelles, misfolded proteins, and protein aggregates which have toxic effects on kidney tubules. By doing so, it promotes cellular hemostasis and survival. Concerning this matter, previous publications have signified the massive accumulation of impaired mitochondria and protein occlusions in mouse renal cells after deficiency of autophagy (Kimura et al., 2011; Takahashi A. et al., 2012) and that activation of autophagy improved mitochondrial function and protected against CP-induced AKI (Zhu et al., 2020). Moreover, Kimura et al. (2012) found that enhancing autophagic flux by IFN-γ alleviated the accumulation of polyubiquitinated peptides, thereby protecting renal tubular cells from CP-induced AKI. iii) Autophagy may disrupt other mechanisms involved in CP nephrotoxicity, particularly apoptosis and oxidative stress. In this regard, Takahashi A. et al. (2012) showed that knockout of Atg5 enhanced p53 stimulation and mitochondrial ROS formation following CP exposure. Similarly, Jiang et al. (2012) found that knockout of Atg7 augmented activation of the p53 cascade. Additionally, it has been reported that inhibition of autophagy accelerated ROS production and apoptosis induced by CP, while its activation reversed these effects (Zhao et al., 2017). Moreover, activation of AMPK attenuated CP-induced AKI by improving mitochondrial function and suppressing the formation of ROS and apoptosis responses caused by CP, all of which effects were abolished upon AMPK inhibition (Zhang J. et al., 2021). Furthermore, restoration of autophagy flux by polydatin ameliorated CP-induced oxidative stress, inflammation, and cell apoptosis, thereby alleviating CP-induced AKI (Li et al., 2022c). iv) Interestingly, several reports have recently documented the pivotal role of the autophagy process in modulating inflammation and ER stress. In response to elevated ER stress during nephrotoxicity, autophagy is strongly activated to mitigate ER stress and counteract tubular cell apoptosis (Pallet et al., 2008). By activating autophagy, Astragaloside IV prevents activation and assembly of NLRP3 inflammasome and reduces the release of pro-inflammatory cytokines in the liver and kidneys injured by CP (Qu et al., 2019). v) Notably, additional reports have also documented the important role of autophagy in preventing renal fibrosis, the main contributor for CKD progression post-AKI. Activation of autophagy participates in elimination of accumulated collagen, thereby attenuating kidney fibrosis and retard the progression of AKI to CKD (Shi et al., 2016). Similarly in the experimental model of CP-induced CKD, the natural flavonoid, farrerol upregulated mitochondrial autophagy, thereby inhibiting inflammation and renal fibrosis induced by CP and closely contributing to CKD (Ma et al., 2021). Along the same line, Shi et al. (2022) recently demonstrated that deficiency of beclin-1, the key molecule of autophagy, promoted renal fibrosis and consequently delayed recovery following renal injury.

#### 5.2.2 Targeting autophagy in CP-induced AKI

The above observations strongly suggest the cytoprotective role of autophagy against CP-induced AKI, and because activation of autophagy by CP is not stable and is relatively retarded over the course of CP injury (Herzog et al., 2012; Li H. et al., 2017; Zhu et al., 2020). Thus, using autophagy inducers could further augment this activation and provide kidney protection during CP chemotherapy. Table 3 summarizes several natural substances and synthetic agents that have been confirmed to exhibit their renoprotective effects through autophagy/mitophagy upregulation in CP-induced AKI.

**TABLE 3:** Summary of renoprotective approaches targeting autophagy/mitophagy in CP-induced AKI.

Compound	Experimental evidence		Autophagy/mitophagy marker change	Molecular mechanism	Effects on CP-induced AKI	Use of autophagy inhibitor or autophagy-deficient mice	Effect of autophagy activator on CP’s chemosensitivity	References
	*In vitro*	*In vivo*						
Retinoic acid (vitamin A derivative)	NRK and OK	Atg5^flox/flox^ or Cagg-Cre and Atg5^flox/flox^:Cagg-Cre mice	↑ LC3-II/I ratio and autophagy puncta, and ↓ p62	n/a	Activation of autophagy alleviates AKI	Attenuated protection	The combination of retinoic acid and CP enhanced CP’s cytotoxicity against gastrointestinal cancer stem cells and re-sensitized resistant lung cancer cells to CP	Najafzadeh et al. (2015); Wu et al. (2020a); MacDonagh et al. (2021)
Metformin (biguanide antihyperglycemic)	NRK-52E	CD1 mice	↑ LC3-II and LC3B puncta and ↓ p-S6	AMPKα activation	Activation of autophagy attenuates CP-induced tubular cell apoptosis and AKI	Abolished protection	Combining metformin with CP significantly strengthened the anti-cancer effect of CP against meningioma both *in vitro* and *in vivo*	Li et al. (2016); Guo et al. (2021)
Penicilliumin B (deep-sea-derived fungi)	HKC-8	C57BL/6 mice	↑ Beclin-1, Atg5, PINK1, autophagic vacuoles, LC3B puncta, LC3B, LC3-II/I ratio, and autophagosome–lysosome fusion, and ↓ p62	AMPK/PGC-1α, and AMPK/mTOR signaling	Activation of autophagy and mitophagy ameliorates impairment in mitochondrial biogenesis and tubular cell apoptosis induced by CP and attenuates AKI	Blocked protection	n/a	Shen et al. (2021)
Lithium (mood stabilizer)	TKPT	C57BL/6 mice	↑ LC3B, LC3BII/I ratio, LC3B puncta, autophagosomes, and ↓ p62	AMPK/mTOR signaling	Activation of autophagy attenuates CP-induced tubular cell apoptosis and AKI	Abrogated protection	The combination of two drugs reversed CP’s chemoresistance in esophageal cancer OE19 and KYSE450 cell lines	O’Donovan et al. (2015); Bao et al. (2019)
Canagliflozin (SGLT2 inhibitor, antihyperglycemic)	HK-2	C57BL/6 mice	↑ LC3B, and LC3B-II	AMPK/mTOR signaling	Activation of autophagy inhibits CP-induced tubular apoptosis and AKI	Abolished protection	Concurrent treatment with canagliflozin and CP neither significantly increased nor decreased antitumor efficacy of CP in lung cancer A549 and colon cancer HCT116 cell lines	Song et al. (2020); Park et al. (2022)
Astragaloside IV (natural triterpenoid saponin)	n/a	Sprague-Dawley rats	↑ LC3 II/I ratio, and ↓ p62	n/a	Activation of autophagy inhibits CP-induced activation of NLRP3 inflammasome and attenuates AKI	n/a	Combined treatment significantly enhances CP’s chemosensitivity against hepatocellular carcinoma both *in vitro* and *in vivo*	Qu et al. (2019), Qu et al. (2020)
Trehalose (natural non-reducing disaccharide)	HK2	C57BL/6 mice	↑ LC3-II, P62, PINK1, Parkin, and LC3/mitochondria colocalization	Activation and nuclear translocation of TFEB	Activation of autophagy and mitophagy inhibit CP-induced mitochondrial dysfunction and apoptosis and protect against AKI	Abrogated protection	Treatment with trehalose significantly counteracts CP-induced apoptosis in Me21 melanoma cells	del Bello et al. (2013); Zhu et al. (2020)
Catalpol (natural iridoid glycoside)	HK-2	Kunming mice	↑ LC3-II/I ratio	AMPKα activation	Activation of mitophagy inhibits mitochondrial membrane potential changes, ROS production, and apoptosis induced by CP and attenuates AKI	Abolished protection	Coadministration of catalpol with CP did not affect the tumoricidal activity of CP in sensitive A549 lung cancer cells but it potentiated its cytotoxicity in resistant A549 cells	Zhang et al. (2021a)
Polydatin (natural precursor of resveratrol)	HK-2	C57BL/6 mice	↑ LC3-II, and autophagolysosome/autophagosome ratio, and ↓ p62	SIRT6 activation	Activation of autophagy reduces oxidative stress, inflammation, and apoptosis induced by CP, and protects against AKI	Abolished protection	Polydatin and CP in combination synergistically reduced tumor size and inhibited lymph node metastasis in *in vivo* model of oral cancer	Mele et al. (2018); Li et al. (2022c)
Berberine (natural isoquinoline alkaloid)	NRK-52E and HKC	C57BL/6	↑ LC3-II/I ratio, PINK1, Parkin, and ↓ p62	PINK1/Parkin signaling	Activation of mitophagy attenuates CP-induced ROS accumulation and AKI	n/a	Co-treatment of berberine and CP markedly enhanced CP’s cytotoxicity against osteosarcoma MG-63, breast cancer MCF-7, and gastric cancer BGC-823 and SGC-7901 cell lines	Zhao et al. (2016); Kou et al. (2020); Qi et al. (2020); Gao et al. (2021)
Suberoylanilide hydroxamic acid and trichostatin A (histone deacetylase inhibitors)	RPTCs	C57BL/6 mice	↑ LC3B-II, LC3B-II turnover, autophagosomes and autolysosomes per cell and per proximal tubule, LC3B-positive puncta per proximal tubule, and autophagic flux rate	AMPK/mTOR signaling	Activation of autophagy inhibits CP-mediated caspase activation, tubular apoptosis and attenuates AKI	Diminished protection	Combining histone deacetylase inhibitors and CP produced synergistic anticancer effects in breast cancer MCF-7 and cholangiocarcinoma KKU-100 and KKU-M214 cell lines	Kim et al. (2003); Asgar et al. (2016); Liu et al. (2018a)
Scutellarin (natural flavonoid)	n/a	C57BL/6 mice	↑ LC3-II/I ratio, Atg7 and ↓ p62	n/a	Activation of autophagy alleviates AKI	n/a	Combined treatment of scutellarin and CP potentiated the anticancer property of CP and ameliorated its resistance in A549 lung cancer cells	Sun et al. (2018a), Sun et al. (2019)
Panax notoginsenoside (Chinese medicinal herb)	n/a	Sprague-Dawley rats	↑ LC3-II, LC3-II/I ratio, Atg5 and Beclin-1	HIF-1α/BNIP3 signaling	Activation of mitophagy attenuates renal damage	n/a	Combined treatment significantly enhanced cytotoxicity of CP in HeLa cells	Zhang et al. (2013); Liu et al. (2015)
Necrostatin-1 (RIPK1 inhibitor)	n/a	C57BL/6 mice	↑ LC3-II, and Beclin-1	n/a	Activation of autophagy alleviates AKI	n/a	In combination, Necrostatin-1 significantly attenuated CP-induced cancer cell apoptosis in KYSE510, but not in KYSE410 esophageal cancer cell lines	Ning et al. (2018); Zhang et al. (2021b)

Abbreviations: CP, cisplatin; AKI, acute kidney injury; NRK, rat renal proximal tubular epithelial cells; OK, opossum kidney cells; LC3-II/I ratio, light chain-II (LC-II)/light chain-I (LC-I) ratio; LC3B, light chain 3 beta; p-S6, phosphorylated S6; AMPKα, AMP-activated protein kinase alpha; HKC-8, human proximal tubular epithelial cells; Beclin-1, Bcl-2-interacting myosin-like coiled-coil protein; Atg5, autophagy-related (Atg) 5; PINK1, PTEN-induced putative kinase protein 1; p-mTOR, phosphorylated mechanistic target of rapamycin; PGC-1α, peroxisome proliferator-activated receptor γ coactivator-1α; TKPT, murine kidney proximal tubular epithelial cells; SGLT2, sodium-glucose co-transporter 2; HK-2, human tubular epithelial cells; NLRP3, NOD-like receptor protein 3, pyrin domain containing; TFEB, transcription factor EB; ROS, reactive oxygen species; SIRT6, sirtuin 6; RPTCs, renal proximal tubular cells; HIF-1α, hypoxia-inducible factor 1-alpha; BNIP3, Bcl-2/adenovirus E1B 19 kDa-interacting protein 3, ↑; increased, ↓; decreased, n/a; not assessed.

## 6 The potential crosstalk between autophagy and necroptosis in CP-induced AKI

Emerging evidence suggests that autophagy and necroptosis can affect each other. To date, only a few studies have provided novel insights into the possible relationship between these two pathways in the kidney. It has been demonstrated that necroptosis molecules may negatively regulate basal autophagy. For example, using the rat CKD model, Shibata et al. (2021) recently suggested that induction of necroptotic signaling in proximal tubular cells may impair the activation of cytoprotective autophagy leading to exacerbation of kidney dysfunction. Moreover, Li R. et al. (2021) showed that RIPK3 inhibits nuclear translocation of TFEB leading to lysosome dysfunction and impairment of autophagic degradation during AKI. Suppression of RIPK3 restored the nuclear translocation of TFEB and attenuated tubular injury *in vitro* and *in vivo*. It has been reported that the deficiency of TFEB increased the mitochondrial ROS production and exacerbate tubular injury during CP renal injury (Lynch et al., 2020). Hence, an interesting question that remains unaddressed is whether inhibitors of necroptosis molecules attenuate CP-induced AKI by controlling lysosomal biogenesis and autophagic flux through TFEB? More interestingly, previous studies also reported that Necrostatin-1, the classical inhibitor of necroptosis alleviated renal injury by increasing the activity of renal autophagy and improving disruption in autophagosome elimination in sepsis- and CP-induced AKI (Dong et al., 2018; Ning et al., 2018). However, whether this renal proautophagic activity is mediated through necroptosis inhibition has not been clarified yet. Therefore, further investigations are required to elucidate autophagy-necroptosis crosstalk in the CP-induced AKI model.

On the other hand, this relationship has been much more investigated in several non-renal cells or tissues. A recent study by Wu X. et al. (2020) reported that increased expression of MLKL enhances western diet-induced liver injury by blocking autophagy through impairment of autophagic flux. Similarly, upon necroptotic stimulation, RIPK3 reduced the activity of autophagy by attenuating autophagic flux in intestinal cells (Otsubo et al., 2020). On the opposite hand, previous studies have also suggested that autophagy may protect against cell necroptosis, while its inhibition may activate and potentiate necroptosis (Matsuzawa-Ishimoto et al., 2017; Zhou et al., 2017; Liu S. et al., 2018; Li et al., 2020; Huang et al., 2021). Recently, Abe et al. (2019) further highlighted this reciprocal regulation by showing that the inhibition of mTORC1 by rapamycin treatment significantly inactivates RIPK1 through inhibitory phosphorylation at Ser320. Inactivation of RIPK1, in turn, stimulates autophagy and represses necroptosis through a TFEB-dependent mechanism. The molecular mechanisms through which autophagy regulates necroptosis are not entirely clear. However, this could be attributed to lysosomal dysfunction, since both necroptotic proteins (RIPK1 & RIPK3) may degrade through the lysosome-dependent pathway and therefore inhibition of lysosomal or autophagy function contributes to their accumulation, thereby triggering necroptosis (Liu S. et al., 2018; Lim et al., 2019). Thus, along with inhibiting apoptosis, these data suggest the ability of autophagy to suppress cell necroptosis which may consider an important mechanism for its pro-survival role.

## 7 Impact of autophagy activation and necroptosis inhibition on anticancer effect of CP

**FIGURE 6:**
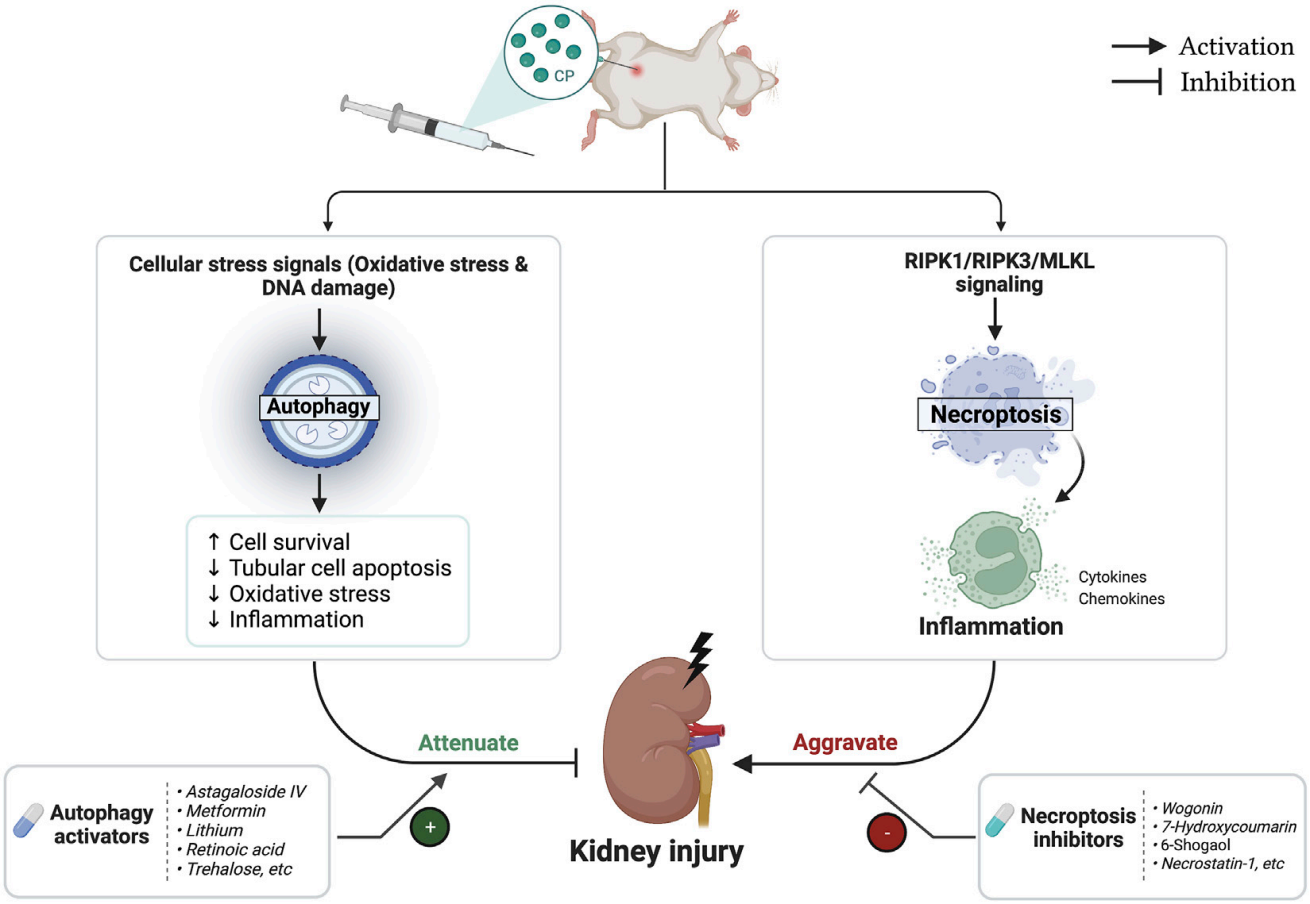
Flowchart representing the role of autophagy and necroptosis pathways during cisplatin (CP)-induced acute kidney injury (AKI).

The biggest challenge that remains is to prevent renal injury associated with CP while maintaining or even enhancing its anti-cancer activity (Sun et al., 2019). Therefore, the main question is could autophagy activation or necroptosis inhibition potentially affect the anticancer activity of CP? Current data indicated that this question still cannot be conclusively answered as both autophagy and necroptosis are considered to have a Janus-faced role in tumorigenesis and cancer treatment. Regarding necroptosis, on one hand, RIPK1 plays a prosurvival role that results in chemoresistance of lung cancer cells. Interestingly, RIPK1 reduction sensitized cancer cells to CP and substantially potentiated its cytotoxicity (Wang et al., 2014a; Wang et al., 2014b; Wang et al., 2017). On the other hand, tumor-suppressing effects of necroptosis have been also reported among other cancer types, especially those resistant to apoptosis. Necroptosis was shown to mediate CP’s cytotoxicity, and its downregulation counteracted the anticancer activities of CP in fibrosarcoma and ovarian cancers (Xu et al., 2017; Zheng et al., 2020). In addition, restoring RIPK3 expression was associated with a better prognosis in patients with hepatocellular and esophageal carcinoma treated with CP chemotherapy (Sun Y. et al., 2018; Zhang B. et al., 2019). Similar to necroptosis, autophagy’s role in CP-induced cytotoxicity is extremely complex and remains a matter of debate, as dual effects of pro-metastatic and anti-metastatic have been reported. Activation of autophagy by CP contributes to its chemoresistance in esophageal cancer (O’Donovan et al., 2011), ovarian cancer (Bao et al., 2015), bladder cancer (Lin et al., 2017), and osteosarcoma (Jiang et al., 2017). In contrast, autophagy induction is also shown to mediate CP chemosensitivity in several other reports. In lung cancer, Sirichanchuen et al. (2012) demonstrated that long-term exposure to CP impaired autophagy leading to CP resistance, and interestingly, upregulation of autophagic response under such condition re-sensitized resistant cells to CP-induced cell death. In addition, autophagic degradation of Exo70 reduced CP efflux, thereby increasing its intracellular storage and cytotoxic activity (Zhao et al., 2021). Moreover, it has been reported that induction of autophagy helps to overcome CP resistance and potentiates its cytotoxic activity against lung cancer (Cheng et al., 2019; López-Plana et al., 2020), and oral cancer (Gao et al., 2022). More importantly, under the context of CP-induced AKI, previous studies have shown inconclusive results. Through upregulation of autophagy, scutellarin ameliorated CP-induced AKI and enhanced its antitumor efficiency against lung cancer (Sun C.-Y. et al., 2018; 2019). Conversely, the proautophagic activity of trehalose conferred protection against CP-induced AKI but also antagonized the antitumor effects of CP (del Bello et al., 2013; Zhu et al., 2020). On the other hand, some other novel autophagy activators like retinoic acid and astragaloside IV, which induce autophagic response in the kidney, and therefore protect against CP-induced AKI (Qu et al., 2019; Wu X. et al., 2020), demonstrated a different response in cancer cells, as they suppressed autophagy to enhance CP’s chemosensitivity (Lai et al., 2020; Abbasi et al., 2022). Altogether, while modulation of autophagy and necroptosis signaling pathways protects kidneys from CP injury (Figure 6), it is still difficult to estimate the effect of this modulation on the clinical response of CP, which is expected to vary depending on tumor type, stage, and cell context as well as the specific individual effect of each modulator. Tables 1, 3 showed the effects of different necroptotic inhibitors and autophagic activators on the anticancer activity of CP chemotherapy.

## 8 Does modulation of autophagy and necroptosis pathways prevent CP-induced toxicities in other organs?

Besides CP-induced nephrotoxicity, administration of CP may induce serious injury in other normal tissues such as the ear, liver, heart, and others (Qi et al., 2019) (Figure 2). Interestingly, several recent publications have indicated the implicated role of necroptosis in CP-mediated ototoxicity (Choi et al., 2019; Ruhl et al., 2019). In Choi et al.’s (2019) study, pretreatment with necroptotic inhibitor, Necrostatin-1 markedly suppressed CP-induced auditory cell death, while the treatment of apoptosis inhibitor, ZVAD did not. Likewise, the protective effect of the autophagy pathway was also reported in CP-induced damage to the liver cells (Qu et al., 2019; Nashar et al., 2021), and cochlear cells (Fang and Xiao, 2014; Yang Q. et al., 2018; Liu et al., 2019; Pang et al., 2019). More Interestingly, trehalose which alleviated CP-induced AKI by activating autophagy (Zhu et al., 2020), recently, its pro-autophagic activity has made it also a potential treatment for CP-induced ototoxicity (Li et al., 2022b). Similarly, finding from other studies demonstrated the ability of the autophagy activator, metformin to attenuate CP-induced ototoxicity, cardiotoxicity, and neurotoxicity primarily through AMPKα activation (Guo et al., 2021; Liang et al., 2021; Nageeb et al., 2022). These data indicate that modulation of autophagy and necroptosis pathways during CP treatment may not only ameliorates CP injury in the kidney but also in other tissues and organs.

## 9 Conclusion and future directions

Nephrotoxicity, especially AKI, is the main serious problem that affects cancer patients treated with CP chemotherapy and often requires cessation of therapy. Therefore, understanding the principal mechanisms underlying this injury would be extremely helpful for the development of effective therapeutic strategies that could substantially help cancer patients to take full efficacy of CP, meanwhile reducing the potential of AKI episodes. In recent years, both autophagy and necroptosis are extensively investigated and their importance in the pathogenesis of CP-induced AKI is increasing at a remarkable pace. Recent *in vitro* and *in vivo* studies have given compelling evidence that modulation of these pathways could provide significant protection against CP-induced AKI. The pharmaceutical industry so far has made significant investments in the development of autophagy and necroptosis modulators, however, none of these agents successfully paved the way for clinical investigation. In the coming years, additional investigations in this area will help the future development of promising modulators with greater efficacy, plasma stability, and specificity. Moreover, given the contribution of various cellular processes in the pathogenesis of CP-induced AKI, using a combination of several modulators or identifying agents that can simultaneously modulate multiple targets may serve as an important strategy for developing future treatments. Importantly, before the clinical translation of any of these modulators, their effects should be adequately examined in tumor-bearing animals to make sure that their renoprotective effects are not compromising the anticancer activity of CP.
